# Honokiol as an α-glucosidase inhibitor

**DOI:** 10.3389/fphar.2024.1425832

**Published:** 2024-06-19

**Authors:** Hua Zhu, Xin Zhong

**Affiliations:** ^1^ School of Chemistry and Chemical Engineering, Mianyang Teacher’s College, Mianyang, China; ^2^ Dean’s Office, Mianyang Teacher’s College, Mianyang, China

**Keywords:** nature product, honokiol, α-glucosidase, inhibition mechanism, inhibitor

## Abstract

Honokiol, a naturally occurring compound from *Magnolia obovata Thunb*., has many biological activities, but its anti-α-glucosidase activity is still unclear. Therefore, we determined its inhibitory effects against α-glucosidase. Activity assays showed that honokiol was a reversible mixed-type inhibitor of α-glucosidase, and its IC_50_ value was 317.11 ± 12.86 μM. Fluorescence results indicated that the binding of honokiol to α-glucosidase caused a reduction in α-glucosidase activity. 3D fluorescence and CD spectra results indicated that the binding of honokiol to α-glucosidase caused conformational change in α-glucosidase. Docking simulated the detailed interactions between honokiol and α-glucosidase, including hydrogen and hydrophobic bonds. All findings showed that honokiol could be used as a natural inhibitor to develop α-glucosidase agents.

## 1 Introduction

Type 2 diabetes mellitus (T2DM) is a growing health concern with increased prevalence (Xu et al., 2020; ElSayed et al., 2023; Zhou et al., 2023). Now, T2DM has become a significant global health issue (Hu et al., 2024; Li et al., 2024). Epidemiological trends indicate that the prevalence of diabetes could reach an alarming 643 million individuals worldwide by 2030 (Song et al., 2022; Lin et al., 2023). The hallmark clinical feature of T2DM is elevated blood glucose levels, or hyperglycemia, which can result in a spectrum of debilitating complications (Jiang et al., 2020; Ding et al., 2021; Xing et al., 2021). Therefore, management of hyperglycemia is a critical aspect for T2DM patients (Hu et al., 2020).

One key characteristic of T2DM is postprandial hyperglycemia, which is intricately linked to the breakdown of carbohydrates (Hameed et al., 2019; Davies et al., 2022). α-Glucosidase, an enzyme present in enterocytes of the small intestine, facilitates the hydrolysis of glycosidic bonds to liberate glucose (Basri et al., 2023; Xiao et al., 2023). The suppression of α-glucosidase activity can thus delay carbohydrate digestion and absorption, leading to a reduction in the postprandial glucose spike (Basri et al., 2023; Wu et al., 2023). This rationale has made the inhibition of α-glucosidase a strategic target for therapeutic interventions to manage postprandial hyperglycemia (Khan et al., 2022; Zhang et al., 2022). Clinically, a number of α-glucosidase inhibitors, including acarbose and voglibose, have been employed to mitigate T2DM (Feng et al., 2024). However, chronic administration of these pharmaceuticals has its drawbacks, which urges people to seek safer and more effective α-glucosidase inhibitors (Lambrinoudaki et al., 2022; Min et al., 2024). Exploration of natural products as a repository for novel therapeutic agents has been a promising avenue (Zhang et al., 2021; Chen et al., 2022; Wang et al., 2022; Zhou et al., 2022). The active constituents have demonstrated a diverse array of pharmacological effects, including anti-oxidant (Sun et al., 2020; Tao et al., 2022; Tang et al., 2023), anti-tumor (Chen et al., 2023; Liang et al., 2023; Song et al., 2023), anti-inflammatory (Sun et al., 2020; Wang et al., 2021; Wang et al., 2022), and anti-tissue damage (Shao et al., 2020; Qi et al., 2022; Ding et al., 2023) properties (Wang et al., 2020; Chen et al., 2022; Zang et al., 2022; He et al., 2023). Moreover, a notable advantage of natural products is their generally lower toxicity profiles (Hao et al., 2022; Mao et al., 2022; Wang et al., 2023), which makes them a preferable source for development of α-glucosidase inhibitors.

**FIGURE 1:**
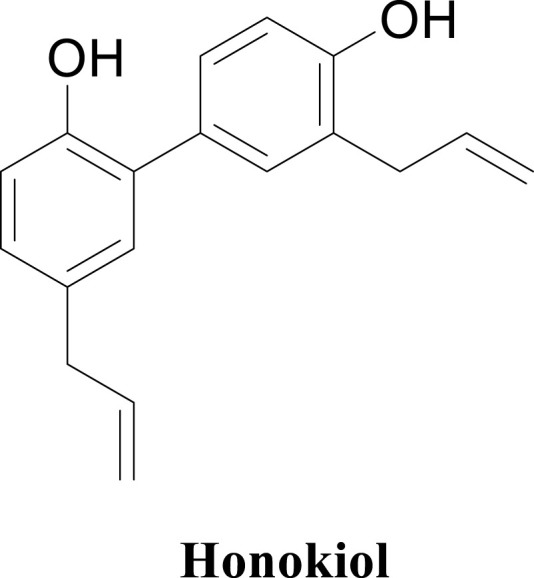
Structure of honokiol.

Honokiol (Figure 1), a naturally occurring compound from *Magnolia obovata* Thunb., has been recognized for its diverse medicinal properties (Zengin et al., 2017; Ma et al., 2023). As a bioactive neolignan, honokiol has demonstrated a range of activities, including anti-cancer, anti-inflammation, and anti-oxidant effects (He et al., 2015; Guo et al., 2021; Niu et al., 2021). In recent research studies, honokiol has garnered significant interest due to its ability to mitigate hyperglycemic conditions, enhance glucose uptake, and inhibit α-glucosidase activity (Bekircan et al., 2015; Pulvirenti et al., 2017; Ahmad et al., 2018). This shows the potential of honokiol as a natural α-glucosidase and hypoglycemic agent.

As far as we know, the detailed inhibitory effects of honokiol on α-glucosidase are still unclear. Hence, the biological activity of honokiol as an α-glucosidase inhibitor was investigated by spectroscopic methods and molecular docking.

## 2 Results and discussion

### 2.1 Inhibitory activity of honokiol on α-glucosidase

**FIGURE 2:**
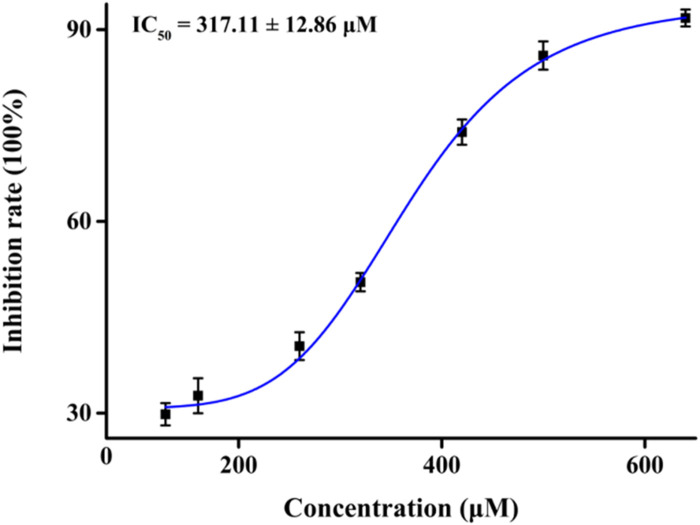
Inhibition of honokiol on α-glucosidase.

First, we assessed the inhibitory activity of honokiol on α-glucosidase, as shown in Figure 2. With an increase in the honokiol concentration, the inhibition rate gradually increased, and its IC_50_ value was calculated to be 317.11 ± 12.86 μM, which was lower than that of acarbose (IC_50_ = 584.51 ± 8.56 μM). The potential inhibitory activity of honokiol on α-glucosidase might make it a natural hypoglycemic agent.

### 2.2 Kinetic study

**FIGURE 3:**
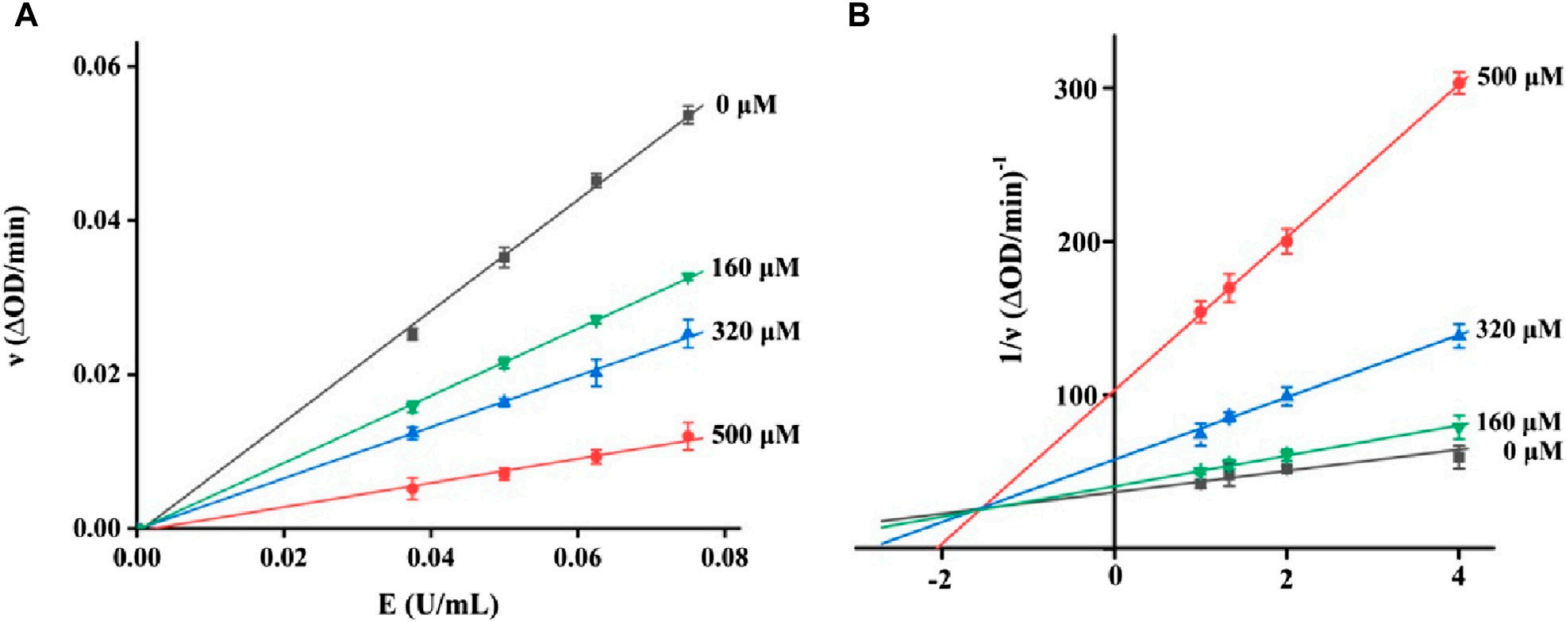
**(A)** Plots of the enzyme reaction rate to enzyme concentration **(B)** Lineweaver–Burk plots of the enzyme reaction rate to the substrate concentration.

It is very important to clarify the inhibition mode of inhibitors against enzymes for understanding the performance of inhibitors. Hence, the kinetics of honokiol on α-glucosidase were studied. In the plots of the enzyme reaction rate to the enzyme concentration under honokiol (Figure 3A), all lines passed the origin point. This indicated honokiol as a reversible α-glucosidase inhibitor. In the Lineweaver–Burk plots of the enzyme reaction rate to substrate concentration under honokiol (Figure 3B), all lines intersected at the second quadrant. Their slope and *Y*-intercept were both changed with honokiol concentration. Therefore, it is evident that honokiol was a mixed-type inhibitor.

**FIGURE 4:**
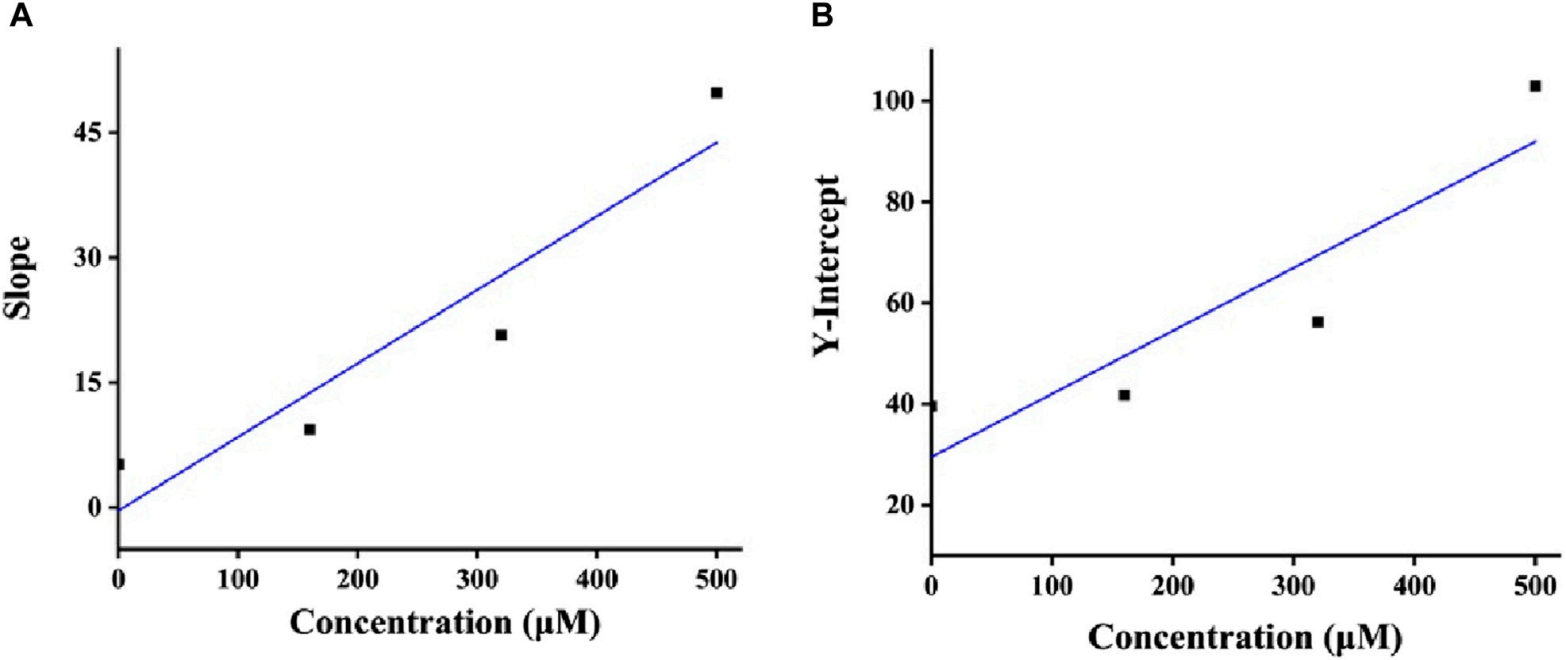
**(A,B)** Ki and Kis plots of honokiol.

As a mixed-type inhibitor, honokiol was determined to have an inhibition constant. The fitting plot of slope and *Y*-intercept *versus* honokiol (Figures 4A,B) yielded Ki and Kis values of 16.03 and 285.22 μM, respectively. The lower Ki indicated that honokiol tended to bind to substrates.

### 2.3 Fluorescence assay

**FIGURE 5:**
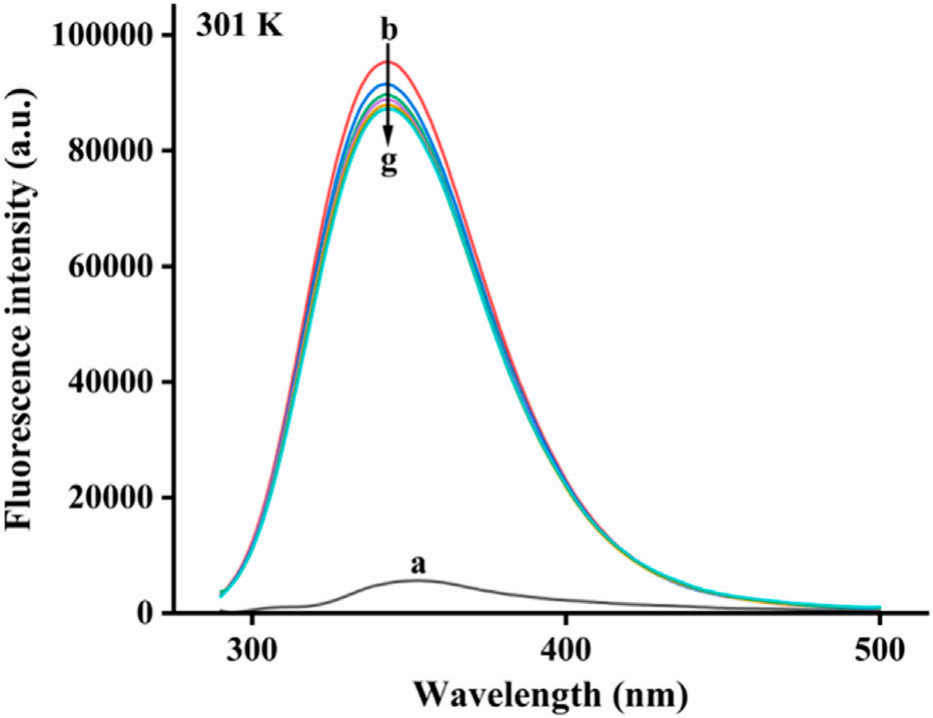
Fluorescence spectra of α-glucosidase binding by honokiol.

Based on the fluorescence characteristics of α-glucosidase, the binding of honokiol to α-glucosidase was studied by fluorescence spectroscopy at 298K. In Figure 5, α-glucosidase presented fluorescence with a characteristic peak at 340 nm, while honokiol had very weak fluorescence at 340 nm. With continuous addition of honokiol, α-glucosidase fluorescence gradually decreased. This phenomenon indicated that there were binding interactions between honokiol and α-glucosidase, which could quench the endogenous fluorescence of α-glucosidase.

**FIGURE 6:**
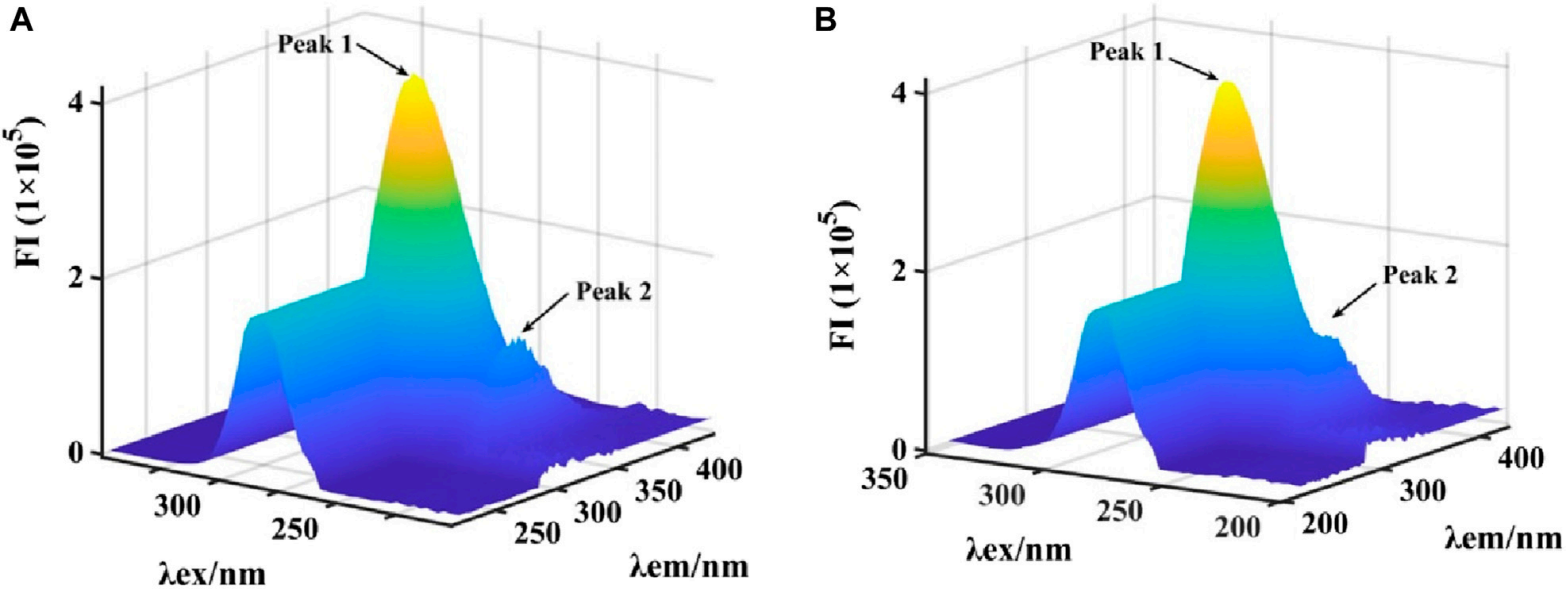
**(A,B)** 3D fluorescence spectra of α-glucosidase binding by honokiol.

Subsequently, the binding of honokiol to α-glucosidase was further described by 3D fluorescence (Figures 6A,B). The 3D fluorescence spectra of α-glucosidase had two characteristic peaks due to intrinsic fluorophores and the backbone, which could be reduced by the addition of honokiol. This result was consistent with that of the fluorescence assay.

### 2.4 CD spectra

**FIGURE 7:**
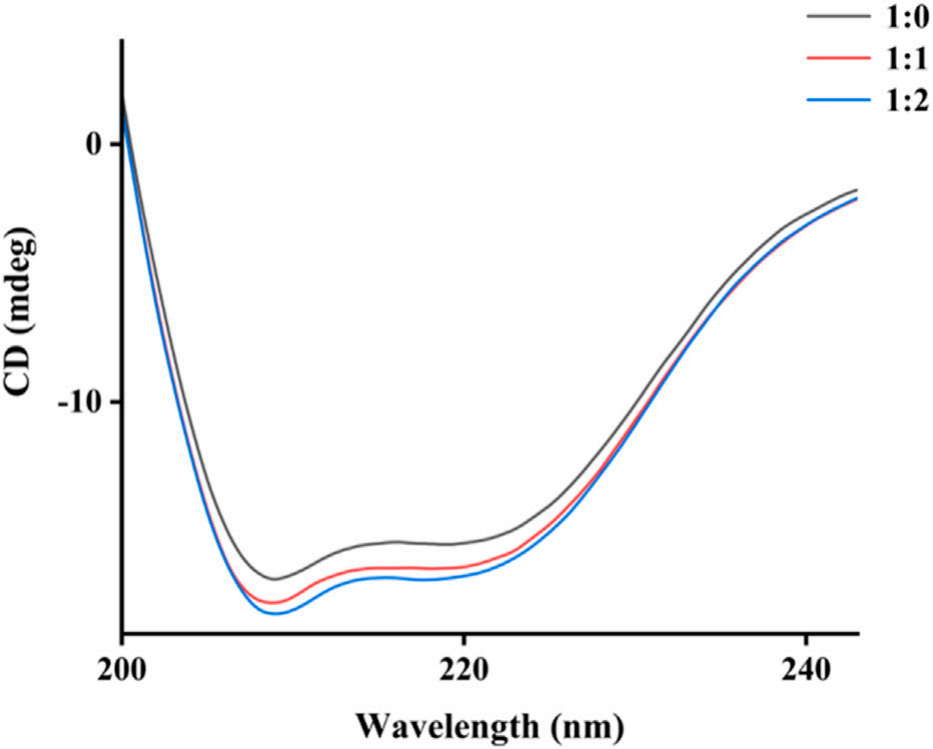
CD spectra of α-glucosidase binding by honokiol.

CD spectra were investigated to evaluate the specific effects of honokiol on α-glucosidase structure. α-Glucosidase showed its own unique CD spectra at 210–222 nm (Figure 7). Honokiol treatment changed the CD spectra of α-glucosidase (Figure 7), which further indicated the binding of honokiol to α-glucosidase. The conformational changes in α-glucosidase were obtained from CD spectral data and showed that honokiol treatment resulted in changes in the α-glucosidase secondary structure content (Table 1). This might be the reason for the inhibition of honokiol on α-glucosidase.

**TABLE 1:** Conformational changes in α-glucosidase by honokiol.

[Enzyme]: [Honokiol]	α-Helix (%)	β-Sheet (%)	β-Turn (%)	Random coil (%)
1: 0	16.7	37.6	20.0	56.2
1: 1	17.0	36.5	19.9	59.4
1: 2	17.2	36.1	19.8	61.5

### 2.5 Molecular docking

**FIGURE 8:**
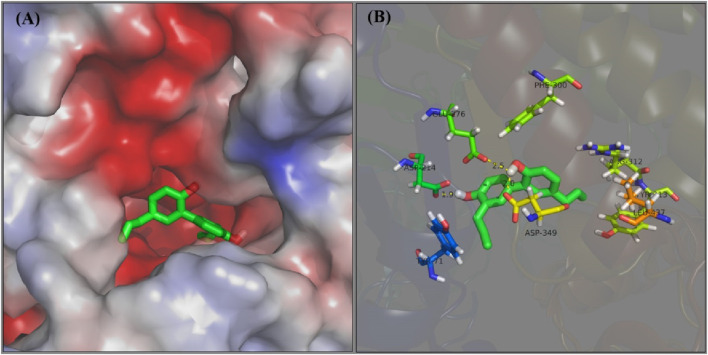
**(A,B)** Docking of honokiol with α-glucosidase in 3D and 2D view.

The docking interaction of honokiol with α-glucosidase was simulated. In a 3D view of docking (Figure 8A), honokiol was bound into the α-glucosidase active pocket, with a binding energy of −4.9 kcal/mol, presumably binding to amino acid residues in the pocket. Further analysis (Figure 8B) found that honokiol formed hydrogen bonds with GLU-276 (2.5 Å), ASP-349 (2.0 Å), and ASP214 (1.9 Å). Moreover, honokiol formed hydrophobic bonds with TYR-71, TYR-313, LEU-437, ARG-312, and PHE300. These main interactions between honokiol and α-glucosidase might be the reason for honokiol’s inhibitory effect on α-glucosidase activity.

## 3 Materials and methods

### 3.1 α-Glucosidase inhibitory activity

α-Glucosidase was dissolved in PBS (pH 6.8), and honokiol was dissolved in DMSO. Honokiol solution and α-glucosidase solution were mixed and incubated for an appropriate time, and then a certain amount of substrate p-nitrophenyl-α-D-galactopyranoside (pNPG) was added. Then, the absorbance of the solution at 405 nm was determined. Then, the α-glucosidase inhibitory effect of honokiol was obtained (Xu et al., 2020; Ali et al., 2023).

### 3.2 Inhibition kinetics

The test procedure for inhibition kinetics followed the same protocol as the α-glucosidase inhibition assay. For enzyme kinetics, the absorbance of a mixture with different concentrations of honokiol and α-glucosidase was recorded. For substrate kinetics, the absorbance of the mixture with different concentrations of honokiol and substrate was recorded (Kaur et al., 2021).

### 3.3 Fluorescence

Fluorescence measurements of α-glucosidase were conducted at an excitation wavelength of 280 nm (Wu et al., 2024). Then, honokiol was added step by step, and the corresponding fluorescence of the mixture was recorded.

### 3.4 3D fluorescence

3D fluorescence spectra of α-glucosidase with/without honokiol were recorded. The concentration of α-glucosidase was 0.1 mg/mL. Honokiol (0.25 μM) was added to α-glucosidase to prepare their mixture.

### 3.5 CD spectra

CD spectra of α-glucosidase with/without honokiol were also recorded. The concentration of α-glucosidase was 0.1 mg/mL. Honokiol was added to α-glucosidase to prepare their mixture. The data were analyzed using CDNN software (Li et al., 2024).

### 3.6 Molecular docking

The docking of honokiol with α-glucosidase was conducted using SYBYL (Deng et al., 2022; Patil et al., 2022). After being imported into the software, the honokiol structure was hydro-treated and charge-treated. Then, the homology model of α-glucosidase was also prepared by hydro-treating and charge-treating. Due to the absence of ligands in the protein, the active pocket of α-glucosidase was produced. Then, the docking of honokiol with α-glucosidase was performed in the default mode.

### 3.7 Statistical analysis

All data were presented as mean ± SD. One-way ANOVA was performed to evaluate the differences between the groups (Zhao et al., 2017; Zhang et al., 2021; Zheng et al., 2021; Sheng et al., 2023). *p* < 0.05 was considered significant.

## 4 Conclusion

As a naturally occurring compound from *Magnolia obovata Thunb*., honokiol was ascertained for its anti-α-glucosidase activity and inhibition mechanism. So we designed experiments to clarify these properties. Activity assays showed that honokiol was a reversible mixed-type inhibitor of α-glucosidase, and its IC_50_ value was 317.11 ± 12.86 μM. Fluorescence, 3D fluorescence, and CD spectra investigations indicated that the binding of honokiol to α-glucosidase caused a reduction in α-glucosidase activity. Docking simulated the detailed interactions between honokiol and α-glucosidase.

## Data Availability

The raw data supporting the conclusion of this article will be made available by the authors, without undue reservation.

## References

[B1] Ahmad S. Nadeem H. Muhammad S. A. Naz S. Imran M. Saeed A. (2018). Synthesis, antimicrobial and α-glucosidase inhibitory potential of Mannich bases of mercapto oxadiazoles and their molecular docking studies. Farmacia 66, 708–717. 10.31925/farmacia.2018.4.22

[B2] Ali A. Ali A. Asati V. Kaya S. Ahsan M. J. (2023). Design, synthesis and anti-hyperglycemic assessments of novel 5-benzylidenethiazolidine-2,4-dione derivatives as PPAR-γ agonist. J. Indian Chem. Soc. 100, 101100. 10.1016/j.jics.2023.101100

[B3] Basri R. Ullah S. Khan A. Mali S. N. Abchir O. Chtita S. (2023). Synthesis, biological evaluation and molecular modelling of 3-Formyl-6-isopropylchromone derived thiosemicarbazones as α-glucosidase inhibitors. Bioorg. Chem. 139, 106739. 10.1016/j.bioorg.2023.106739 37478545

[B4] Bekircan O. Ülker S. Menteşe E. (2015). Synthesis of some novel heterocylic compounds derived from 2-[3-(4-chlorophenyl)-5-(4-methoxybenzyl)-4H-1, 2, 4-triazol-4-yl] acetohydrazide and investigation of their lipase and α-glucosidase inhibition. J. Enzyme Inhib. Med. Chem. 30, 1002–1009. 10.3109/14756366.2014.1003213 25640970

[B5] Chen J. Cao D. Jiang S. Liu X. Pan W. Cui H. (2022). Triterpenoid saponins from Ilex pubescens promote blood circulation in blood stasis syndrome by regulating sphingolipid metabolism and the PI3K/AKT/eNOS signaling pathway. Phytomedicine 104, 154242. 10.1016/j.phymed.2022.154242 35728385

[B6] Chen W. Zhang S. Pan S. Wang Z. Xu J. Sheng X. (2022). α-Mangostin treats early-stage adjuvant-induced arthritis of rat by regulating the CAP-SIRT1 pathway in macrophages. Drug Des. Dev. Ther. 16, 509–520. 10.2147/DDDT.S348836 PMC8893152 35250263

[B7] Chen Y. Yin S. Liu R. Yang Y. Wu Q. Lin W. (2023). β-Sitosterol activates autophagy to inhibit the development of hepatocellular carcinoma by regulating the complement C5a receptor 1/alpha fetoprotein axis. Eur. J. Pharmacol. 957, 175983. 10.1016/j.ejphar.2023.175983 37598926

[B8] Davies M. J. Aroda V. R. Collins B. S. Gabbay R. A. Green J. Maruthur N. M. (2022). Management of hyperglycaemia in type 2 diabetes, 2022. A consensus report by the American diabetes association (ADA) and the European association for the study of diabetes (EASD). Diabetologia 65, 1925–1966. 10.1007/s00125-022-05787-2 36151309 PMC9510507

[B9] Deng X. Ke J. Zheng Y. Li D. Zhang K. Zheng X. (2022). Synthesis and bioactivities evaluation of oleanolic acid oxime ester derivatives as α-glucosidase and a-amylase inhibitors. J. Enzyme Inhib. 37, 451–461. 10.1080/14756366.2021.2018682 PMC8757604 35012401

[B10] Ding D. Shen X. Yu L. Zheng Y. Liu Y. Wang W. (2023). Timosaponin BII inhibits TGF‐β mediated epithelial‐mesenchymal transition through Smad-dependent pathway during pulmonary fibrosis. Phytother. Res. 37, 2787–2799. 10.1002/ptr.7774 36807664

[B11] Ding M. Tang Z. Liu W. Shao T. Yuan P. Chen K. (2021). Burdock fructooligosaccharide attenuates high glucose-induced apoptosis and oxidative stress injury in renal tubular epithelial cells. Front. Pharmacol. 12, 784187. 10.3389/fphar.2021.784187 34955856 PMC8695902

[B12] ElSayed N. A. Aleppo G. Aroda V. R. Bannuru R. R. Brown F. M. Bruemmer D. (2023). 2. Classification and diagnosis of diabetes: standards of care in diabetes-2023. Diabetes Care 46, S19–S40. 10.2337/dc23-S002 36507649 PMC9810477

[B13] Feng M. Liang B. Sun J. Min X. Wang S. Lu Y. (2024). Synthesis, anti-α-glucosidase activity, inhibition interaction, and anti-diabetic activity of novel cryptolepine derivatives. J. Mol. Struct. 1310, 138311. 10.1016/j.molstruc.2024.138311

[B14] Guo Y. Hou E. Wen T. Yan X. Han M. Bai L. (2021). Development of membrane-active honokiol/magnolol amphiphiles as potent antibacterial agents against methicillin-resistant *Staphylococcus aureus* (MRSA). J. Med. Chem. 64, 12903–12916. 10.1021/acs.jmedchem.1c01073 34432450

[B15] Hameed S. Seraj F. Rafique R. Chigurupati S. Wadood A. Ur Rehman A. (2019). Synthesis of benzotria-zoles derivatives and their dual potential as α-amylase and α-glucosidase inhibitors *in vitro*: structure-activity relationship, molecular docking, and kinetic studies. Eur. J. Med. Chem. 183, 111677. 10.1016/j.ejmech.2019.111677 31514061

[B16] Hao J. Bei J. Li Z. Han M. Ma B. Ma P. (2022). Qing`e pill inhibits osteoblast ferroptosis via ATM serine/threonine kinase (ATM) and the PI3K/AKT pathway in primary osteoporosis. Front. Pharmacol. 13, 902102. 10.3389/fphar.2022.902102 35865965 PMC9294279

[B17] He L. Fan T. Hu J. Zhang L. (2015). Polyethylene glycol-based ultrasound-assisted extraction of magnolol and honokiol from Cortex Magnoliae Officinalis. Nat. Prod. Res. 29, 31–36. 10.1080/14786419.2014.955800 25204856

[B18] He X. Liu G. Chen X. Wang Y. Liu R. Wang C. (2023). Pharmacokinetic and pharmacodynamic interactions between henagliflozin, a novel selective SGLT-2 inhibitor, and warfarin in healthy Chinese subjects. Clin. Ther. 45, 655–661. 10.1016/j.clinthera.2023.06.002 37451912

[B19] Hu C. Liang B. Sun J. Li J. Xiong Z. Wang S. (2024). Synthesis and biological evaluation of indole derivatives containing thiazolidine-2,4-dione as α-glucosidase inhibitors with antidiabetic activity. Eur. J. Med. Chem. 264, 115957. 10.1016/j.ejmech.2023.115957 38029465

[B20] Hu C. Wang Y. Xi Y. Yao X. (2020). Dapagliflozin therapy curative effect observation on nonalcoholic fatty liver disease in patients with type 2 diabetes mellitus. Indian J. Pharm. Sci. 82, 122–129. 10.36468/pharmaceutical-sciences.spl.155

[B21] Jiang Y. Li W. Wang J. Wang G. (2020). Cardiac dysfunction is attenuated by ginkgolide B *via* reducing oxidative stress and fibrosis in diabetic rats. Iran. J. Basic Med. Sci. 23, 1078–1084. 10.22038/ijbms.2020.44210.10358 PMC7478264 32952955

[B22] Kaur R. Kumar R. Dogra N. Kumar A. Yadav A. K. Kumar M. (2021). Synthesis and studies of thiazolidinedione-isatin hybrids as α-glucosidase inhibitors for management of diabetes. Future Med. Chem. 13, 457–485. 10.4155/fmc-2020-0022 33506699

[B23] Khan S. Ullah H. Rahim F. Nawaz M. Hussain R. Rasheed L. (2022). Synthesis, *in vitro* α-amylase, α-glucosidase activities and molecular docking study of new benzimidazole bearing thiazolidinone derivatives. J. Mol. Struct. 1269, 133812. 10.1016/j.molstruc.2022.133812

[B24] Lambrinoudaki I. Paschou S. A. Armeni E. Goulis D. G. (2022). The interplay between diabetes mellitus and menopause: clinical implications. Nat. Rev. Endocrinol. 18, 608–622. 10.1038/s41574-022-00708-0 35798847

[B25] Li M. Li H. Min X. Sun J. Liang B. Xu L. (2024). Identification of 1,3,4-thiadiazolyl-containing thiazolidine-2,4-dione derivatives as novel PTP1B inhibitors with antidiabetic activity. J. Med. Chem. 67, 8406–8419. 10.1021/acs.jmedchem.4c00676 38723203

[B26] Li M. Sun J. Liang B. Min X. Hu J. Wu R. (2024). Thiazolidine-2,4-dione derivatives as potential α-glucosidase inhibitors: synthesis, inhibitory activity, binding interaction and hypoglycemic activity. Bioorg. Chem. 144, 107177. 10.1016/j.bioorg.2024.107177 38335756

[B27] Liang T. Wang F. Elhassan R. Cheng Y. Tang X. Chen W. (2023). Targeting histone deacetylases for cancer therapy: trends and challenges. Acta Pharm. Sin. B 13, 2425–2463. 10.1016/j.apsb.2023.02.007 37425042 PMC10326266

[B28] Lin J. Xiao D. Lu L. Liang B. Xiong Z. Xu X. (2023). New β-carboline derivatives as potential α-glucosidase inhibitor: synthesis and biological activity evaluation. J. Mol. Struct. 1283, 135279. 10.1016/j.molstruc.2023.135279

[B29] Ma M. Wei N. Yang J. Ding T. Song A. Chen L. (2023). Schisandrin B promotes senescence of activated hepatic stellate cell via NCOA4-mediated ferritinophagy. Pharm. Biol. 61, 621–629. 10.1080/13880209.2023.2189908 37010139 PMC10071970

[B30] Mao Y. Xu R. Li Y. Jiang F. (2022). Baicalin ameliorates preeclampsia *in vitro* by regulating the miRNA-19a/PTEN axis. Lat. Am. J. Pharm. 41, 2254–2265.

[B31] Min X. Guo S. Lu Y. Xu X. (2024). Investigation on the inhibition mechanism and binding behavior of cryptolepine to α-glucosidase and its hypoglycemic activity by multi-spectroscopic method. J. Lumin. 269, 120437. 10.1016/j.jlumin.2024.120437

[B32] Niu L. Hou Y. Jiang M. Bai G. (2021). The rich pharmacological activities of Magnolia officinalis and secondary effects based on significant intestinal contributions. J. Ethnopharmacol. 281, 114524. 10.1016/j.jep.2021.114524 34400262

[B33] Patil V. M. Tilekar K. N. Upadhyay N. M. Ramaa C. S. (2022). Synthesis, *in-vitro* evaluation and molecular docking study of N-substituted thiazolidinediones as α-glucosidase inhibitors. ChemistrySelect 7, e202103848. 10.1002/slct.202103848

[B34] Pulvirenti L. Muccilli V. Cardullo N. Spatafora C. Tringali C. (2017). Chemoenzymatic synthesis and α-glucosidase inhibitory activity of dimeric neolignans inspired by magnolol. J. Nat. Prod. 80, 1648–1657. 10.1021/acs.jnatprod.7b00250 28497968

[B35] Qi X. Zheng S. Ma M. Lian N. Wang H. Chen L. (2022). Curcumol suppresses CCF-mediated hepatocyte senescence through blocking LC3B-Lamin B1 interaction in alcoholic fatty liver disease. Front. Pharmacol. 13, 912825. 10.3389/fphar.2022.912825 35837283 PMC9273900

[B36] Shao X. Li B. Shen J. Wang Q. Chen S. Jiang X. (2020). Ghrelin alleviates traumatic brain injury-induced acute lung injury through pyroptosis/NF-κB pathway. Int. Immunopharmacol. 79, 106175. 10.1016/j.intimp.2019.106175 31918060

[B37] Sheng B. Lai N. Tao T. Chen X. Gao S. Zhu Q. (2023). Diagnosis potential of subarachnoid hemorrhage using miRNA signatures isolated from plasma-derived extracellular vesicles. Front. Pharmacol. 14, 1090389. 10.3389/fphar.2023.1090389 36860299 PMC9968748

[B38] Song A. Ding T. Wei N. Yang J. Ma M. Zheng S. (2023). Schisandrin B induces HepG2 cells pyroptosis by activating NK cells mediated anti-tumor immunity. Toxicol. Appl. Pharmacol. 472, 116574. 10.1016/j.taap.2023.116574 37271225

[B39] Song J. Xu H. Zhang W. Yang C. Li L. Luan J. (2022). Impact of solute carrier family 47 member 1 gene polymorphism detection on therapeutic effect of diabetes. Int. J. Pharmacol. 18, 398–406. 10.3923/ijp.2022.398.406

[B40] Sun S. Li S. Du Y. Wu C. Zhang M. Li J. (2020a). Anti-inflammatory effects of the root, stem and leaf extracts of *Chloranthus serratus* on adjuvant-induced arthritis in rats. Pharm. Biol. 58, 528–537. 10.1080/13880209.2020.1767159 32503379 PMC8641675

[B41] Sun S. Wang Y. Du Y. Sun Q. He L. Zhu E. (2020b). Oxidative stress-mediated hepatotoxicity in rats induced by ethanol extracts of different parts of Chloranthus serratus. Pharm. Biol. 58, 1277–1289. 10.1080/13880209.2020.1859552 33355514 PMC7759245

[B42] Tang Z. Zhang M. Gao L. Bao Y. Li P. Wang M. (2023). Optimal extraction of polysaccharides from Stevia rebaudiana roots for protection against hydrogen peroxide-induced oxidative damage in RAW264.7 cells. Nat. Prod. Res., 2263905. 10.1080/14786419.2023.2263905 37791599

[B43] Tao Z. Li T. Wei S. (2022). Silymarin prevents iron overload induced bone loss by inhibiting oxidative stress in an ovariectomized animal model. Chem. Biol. Interact. 366, 110168. 10.1016/j.cbi.2022.110168 36087815

[B44] Wang H. Chen Y. Wang L. Liu Q. Yang S. Wang C. (2023). Advancing herbal medicine: enhancing product quality and safety through robust quality control practices. Front. Pharmacol. 14, 1265178. 10.3389/fphar.2023.1265178 37818188 PMC10561302

[B45] Wang J. Li D. Ni W. Qin X. Liu H. Yu L. L. (2020). Molecular networking uncovers steroidal saponins of Paris tengchongensis. Fitoterapia 145, 104629. 10.1016/j.fitote.2020.104629 32428563

[B46] Wang R. Li D. Hu Y. Liao Q. Jiang T. Olatunji O. J. (2021). Qing-Luo-Yin alleviated monocytes/macrophages-mediated inflammation in rats with adjuvant-induced arthritis by disrupting their interaction with (Pre)-Adipocytes through PPAR-γ signaling. Drug. Des. Dev. Ther. 15, 3105–3118. 10.2147/DDDT.S320599 PMC8291661 34295151

[B47] Wang X. Zhou D. Zhou W. Liu J. Xue Q. Huang Y. (2022). Clematichinenoside AR inhibits the pathology of rheumatoid arthritis by blocking the circPTN/miR-145-5p/FZD4 signal axis. Int. Immunopharmacol. 113, 109376. 10.1016/j.intimp.2022.109376 36279670

[B48] Wang Y. Wu H. Han Z. Sheng H. Wu Y. Wang Y. (2022). Guhong injection promotes post-stroke functional recovery via attenuating cortical inflammation and apoptosis in subacute stage of ischemic stroke. Phytomedicine 99, 154034. 10.1016/j.phymed.2022.154034 35276592

[B49] Wu S. Tang J. Zhou Y. Xu X. Zhang H. Wang S. (2024). α-Glucosidase inhibition research of derivatives based on 2β-acetoxyferruginol scaffold excluding acetic acid group. Chin. J. Org. Chem. 44, 613–621. 10.6023/cjoc202307027

[B50] Wu X. Zhu W. Lu L. Hu C. Zheng Y. Zhang X. (2023). Synthesis and anti-a-glucosidase activity evaluation of betulinic acid derivatives. Arab. J. Chem. 16, 104659. 10.1016/j.arabjc.2023.104659

[B51] Xiao D. Lu L. Liang B. Xiong Z. Xu X. Chen W. (2023). Identification of 1,3,4-oxadiazolyl-containing β-carboline derivatives as novel α-glucosidase inhibitors with antidiabetic activity. Eur. J. Med. Chem. 261, 115795. 10.1016/j.ejmech.2023.115795 37688939

[B52] Xing Y. Liu B. Wan S. Cheng Y. Zhou S. Sun Y. (2021). A SGLT2 inhibitor dapagliflozin alleviates diabetic cardiomyopathy by suppressing high glucose-induced oxidative stress *in vivo* and *in vitro* . Front. Pharmacol. 12, 708177. 10.3389/fphar.2021.708177 34322029 PMC8311522

[B53] Xu S. He L. Ding K. Zhang L. Xu X. Wang S. (2020). Tanshinone IIA ameliorates streptozotocin-induced diabetic nephropathy, partly by attenuating PERK pathway-induced fibrosis. Drug Des. Dev. Ther. 14, 5773–5782. 10.2147/DDDT.S257734 PMC7780857 33408464

[B54] Xu X. Deng X. Chen J. Liang Q. Zhang K. Li D. (2020). Synthesis and biological evaluation of coumarin derivatives as aglucosidase Inhibitors. Eur. J. Med. Chem. 189, 112013. 10.1016/j.ejmech.2019.112013 31972390

[B55] Zang L. Xu H. Huanh C. Wang C. Wang R. Chen R. (2022). A link between chemical structure and biological activity in triterpenoids. Recent Pat. Anti-Canc 17, 145–161. 10.2174/1574892816666210512031635 33982656

[B56] Zengin G. Locatelli M. Stefanucci A. Macedonio G. Mollica A. Mirzaie S. (2017). Chemical characterization, antioxidant properties, anti-inflammatory activity, and enzyme inhibition of Ipomoea batatas L. leaf extracts. Int. J. Food Prop. 20, 1–13. 10.1080/10942912.2017.1357127

[B57] Zhang X. Lu Y. Li W. Tao T. Peng L. Wang W. (2021). Astaxanthin ameliorates oxidative stress and neuronal apoptosis via SIRT1/NRF2/Prx2/ASK1/p38 after traumatic brain injury in mice. Brit. J. Pharmacol. 178, 1114–1132. 10.1111/bph.15346 33326114

[B58] Zhang X. Zheng Y. Hu C. Wu X. Lin J. Xiong Z. (2022). Synthesis and biological evaluation of coumarin derivatives containing oxime ester as a-glucosidase inhibitors. Arab. J. Chem. 15, 104072. 10.1016/j.arabjc.2022.104072

[B59] Zhang Y. Zhang X. Li H. Zhou T. Zhou A. Zhong Z. (2021). Antidepressant-like effects of helicid on a chronic unpredictable mild stress-induced depression rat model: inhibiting the IKK/IκBα/NF-κB pathway through NCALD to reduce inflammation. Int. Immunopharmacol. 93, 107165. 10.1016/j.intimp.2020.107165 33578182

[B60] Zhao S. Ma L. Chu Z. Xu H. Wu W. Liu F. (2017). Regulation of microglial activation in stroke. Acta Pharmacol. Sin. 38, 445–458. 10.1038/aps.2016.162 28260801 PMC5386316

[B61] Zheng Y. Ding W. Zhang T. Zhao Z. Wang R. Li Z. (2021). Antimony-induced astrocyte activation via mitogen-activated protein kinase activation-dependent CREB phosphorylation. Toxicol. Lett. 352, 9–16. 10.1016/j.toxlet.2021.09.006 34571074

[B62] Zhou Y. Liu L. Xiang R. Bu X. Qin G. Dai J. (2023). Arctigenin mitigates insulin resistance by modulating the IRS2/GLUT4 pathway via TLR4 in type 2 diabetes mellitus mice. Int. Immunopharmacol. 114, 109529. 10.1016/j.intimp.2022.109529 36481528

[B63] Zhou Y. Xiang R. Qin G. Ji B. Yang S. Wang G. (2022). Xanthones from *Securidaca inappendiculata Hassk.* attenuate collagen-induced arthritis in rats by inhibiting the nicotinamide phosphoribosyltransferase/glycolysis pathway and macrophage polarization. Int. Immunopharmacol. 111, 109137. 10.1016/j.intimp.2022.109137 36001918

